# Management of comminuted patellar fracture fixation using modified cerclage wiring

**DOI:** 10.1186/s13018-019-1385-5

**Published:** 2019-10-17

**Authors:** Yangyang Sun, Kuisheng Sheng, Qinghu Li, Dawei Wang, Dongsheng Zhou

**Affiliations:** 1 Department of Orthopaedic Surgery, Shandong Provincial Hospital Affiliated to Shandong University, 324 Jingwuweiqi Road, Jinan, Shandong People’s Republic of China; 2 Department of Orthopaedic Surgery, Rizhao Traditional Chinese Medical Hospital, Rizhao, Shandong People’s Republic of China

**Keywords:** Patellar fracture, Comminuted fracture, Cerclage wiring

## Abstract

**Background:**

Although there are several different kinds of fixation techniques for displaced comminuted patellar fracture, the treatment remains a challenge for orthopaedic surgeons. The purpose of this study is to evaluate the effectiveness and safety of a fixation technique for comminuted patellar fracture fixation using modified cerclage wiring.

**Methods:**

From February 2016 to April 2018, 38 cases of simple unilateral closed comminuted patellar fracture were treated by modified cerclage wiring. Among these cases, 16 patients were males and 22 were females, aged 23–68 years (average 40.4 ± 9.1 years). Comminuted patellar fractures were classified according to the AO/OTA classification: 10 cases were type 34-C2 (three fragments), 28 cases were type 34-C3 (more than three fragments). Postoperative complications including loosening of internal fixation, fragment re-displacement, nonunion, infection, breakage of internal fixation and traumatic osteoarthritis were assessed. The clinical results after operation were evaluated by the clinical grading scales of Böstman including range of movement, pain, work, atrophy, assistance in walking, effusion, giving way, and stair-climbing during follow-up.

**Results:**

A total of 38 patients were followed up for 6–36 months (mean time 16.1 ± 5.8 months). The bone union radiographically occurred at approximately 2.5–3.5 months (mean time 2.92 ± 0.25 months). No postoperative complications, such as infection, dislocation, breakage of the implants, painful hardware, and post-traumatic osteoarthritis, were observed. According to the clinical grading scales of Böstman, satisfactory results were obtained, and the mean score at the final follow-up was 28.7 (range 20–30) points. Thirty-two patients (84.2%) with excellent results had a mean score of 29.5 ± 0.7 (range 28–30) points, and six patients (15.8%) with good results had a mean score of 24.5 ± 2.2 (range 20–27) points. The patients with excellent and good scores had active flexion of 130° (110–140).

**Conclusions:**

Modified cerclage wiring can effectively treat comminuted patellar fracture and offers a new strategy resulting in satisfactory results without obvious complications.

## Introduction

Displaced comminuted patellar fracture requires surgical treatment [1, 2]. The purpose of surgical treatment is to restore the patellar articular surface and the disrupted knee extensor mechanism [3]. Patellar comminuted fracture is a great challenge for clinical orthopaedic surgeons. The main challenge is that sometimes, it is difficult to obtain anatomical reduction and rigid internal fixation, resulting in poor functional outcome.

At present, the treatment methods of patellar comminuted fracture include the following: circumferential cerclage wire fixation [2], modified tension band fixation [4, 5], nickel-titanium patella concentrator [6], cable-pin system [7], titanium cable cerclage [8], plate and screw fixation [9] and partial or total patellectomy [2, 10]. Partial or total patellectomy results in the destruction of the extensor mechanism and normal patellofemoral joint contact surface, which reduces knee joint function [1, 10, 11]. Therefore, this treatment can only be used as a remedy when the comminuted bone cannot be reduced. Open reduction and internal fixation is the first choice for the treatment of comminuted patellar fracture [12]. Through internal fixation technology, the fragments can be fixed stably to carry out the early functional exercise of the knee. Modified tension band fixation has a good effect on simple transverse patellar fracture [13], and the curative effect on the comminuted patella remains to be discussed. Plate and screw fixations are used for the treatment of patellar fracture, but biomechanical studies are mainly used for the treatment of transverse patellar fracture or inferior patellar fracture [14]. Circumferential cerclage wire fixation is suitable for the treatment of a comminuted patellar fracture. Biomechanical results showed that the stability of cerclage wire fixation was significantly worse than that of tension band and modified tension band [15]. For fracture fragments, the fixation strength was not enough to resist the contraction of quadriceps femoris or the tension caused by knee flexion. Fracture fragments were prone to move, so the patients could not exercise in the early stage. External fixation was often needed for 6–8 weeks or longer, and quadriceps femoris atrophy was easily formed. Early complications, such as contraction and stiffness of knee joint, contribute to poor long-term recovery of joint function. Some scholars reported that two or more fixation methods were combined to treat patellar comminuted fracture, such as circumferential cerclage wire fixation combined with modified tension band [16], non-absorbable suture cerclage combined with nickel-titanium patellar concentrator [17] and headless compression and wiring technique [18].

We propose a new strategy for the treatment of comminuted patellar fracture with modified cerclage wiring fixation. The objective of this study was to evaluate the effect of modified cerclage wiring fixation through radiographic, clinical and functional outcome data on patients with comminuted patellar fracture. Postoperative complications, fracture healing and the clinical grading scales of Böstman were the main outcome indicators. We hypothesize that modified cerclage wiring fixation can effectively fix the comminuted patellar fracture, provide rigid fixation for early functional exercise, predict fracture healing, and have good function and a low incidence of complications related to internal fixation.

## Materials and methods

### General information

The study protocol was approved by the Ethics Committee of Shandong Provincial Hospital affiliated with Shandong University. Written informed consent was obtained from all the patients included in this study. From February 2016 to April 2018, 38 cases of simple unilateral closed comminuted patellar fracture were treated by modified cerclage wiring. There were 16 males and 22 females, aged 23–68 years (average 40.4 ± 9.1 years), 21 left patella and 17 right patella. In the injury mechanism, 12 cases were of traffic accident injury, 20 cases of tumble and 6 cases of fall. According to the AO/OTA classification [19], 10 cases were type 34-C2 (three fragments) and 28 cases were type 34-C3 (more than three fragments). The time from injury to operation was 2–5 days (3.2 ± 1.9 days).

### Surgical procedures

**Fig. 1:**
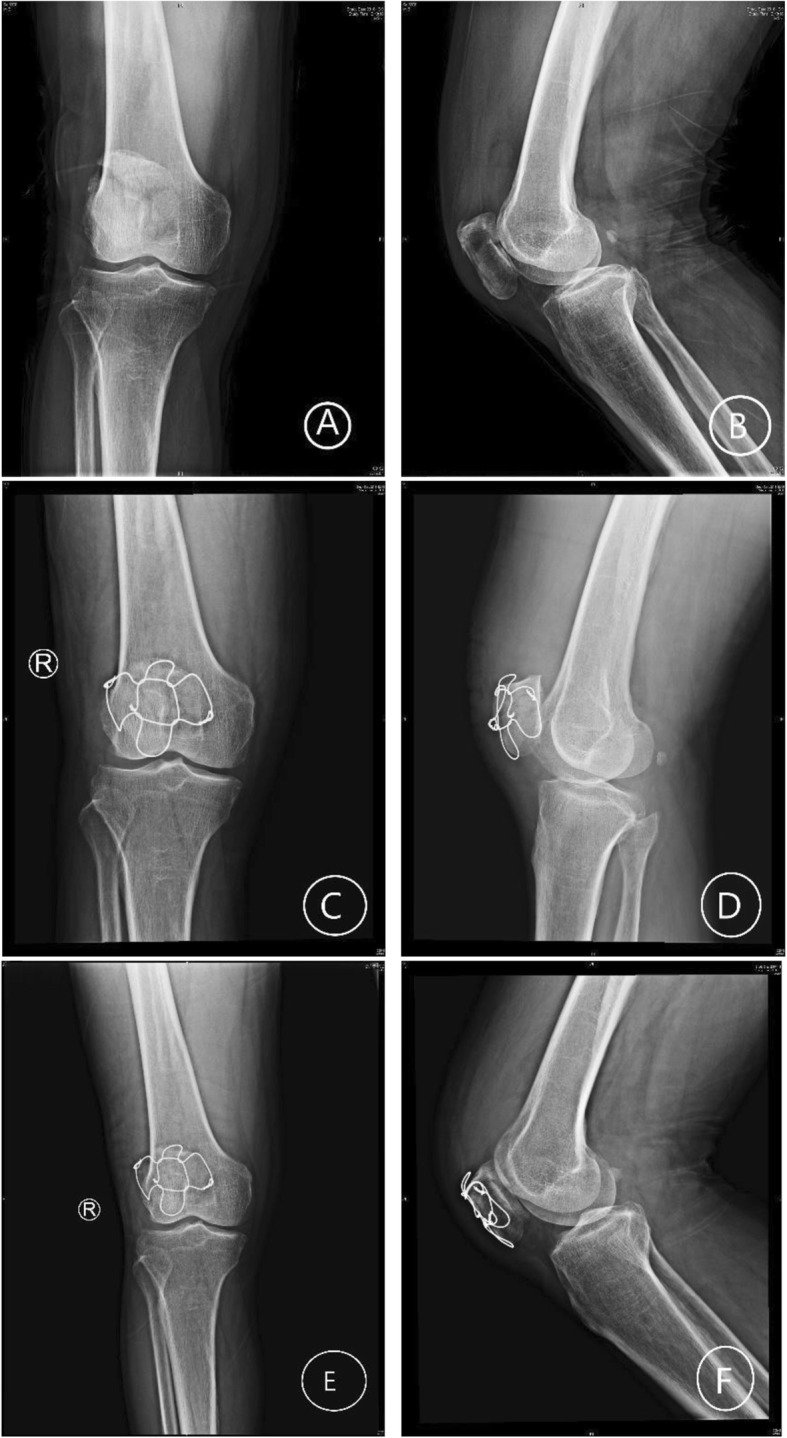
A 36-year-old female with severely comminuted patellar fracture (34-C3). **a**, **b** X-ray before surgery. **c**, **d** Post-fixation with modified cerclage wiring. **e**, **f** 3 months after surgery

**Fig. 2:**
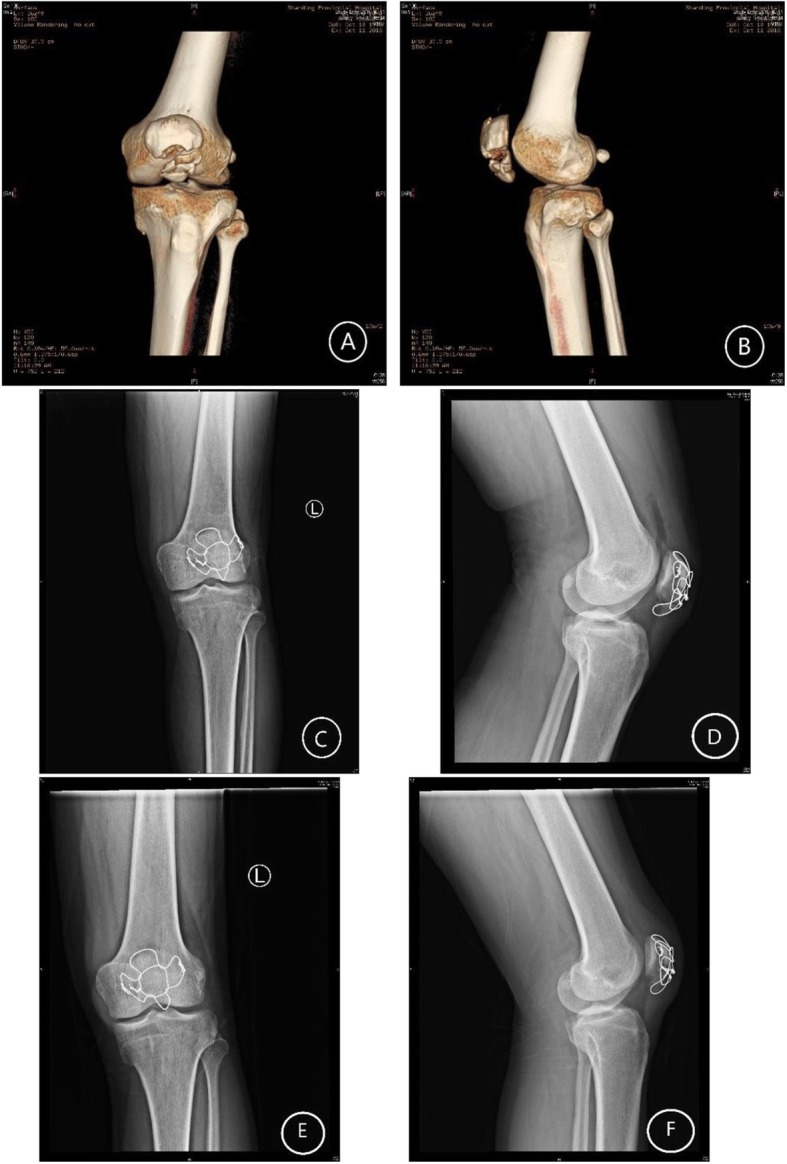
A 39-year-old male with comminuted patellar fracture. **a**, **b** CT before surgery. **c**, **d** Post-fixation with modified cerclage wiring. **e**, **f** 3 months after surgery

**Fig. 3:**
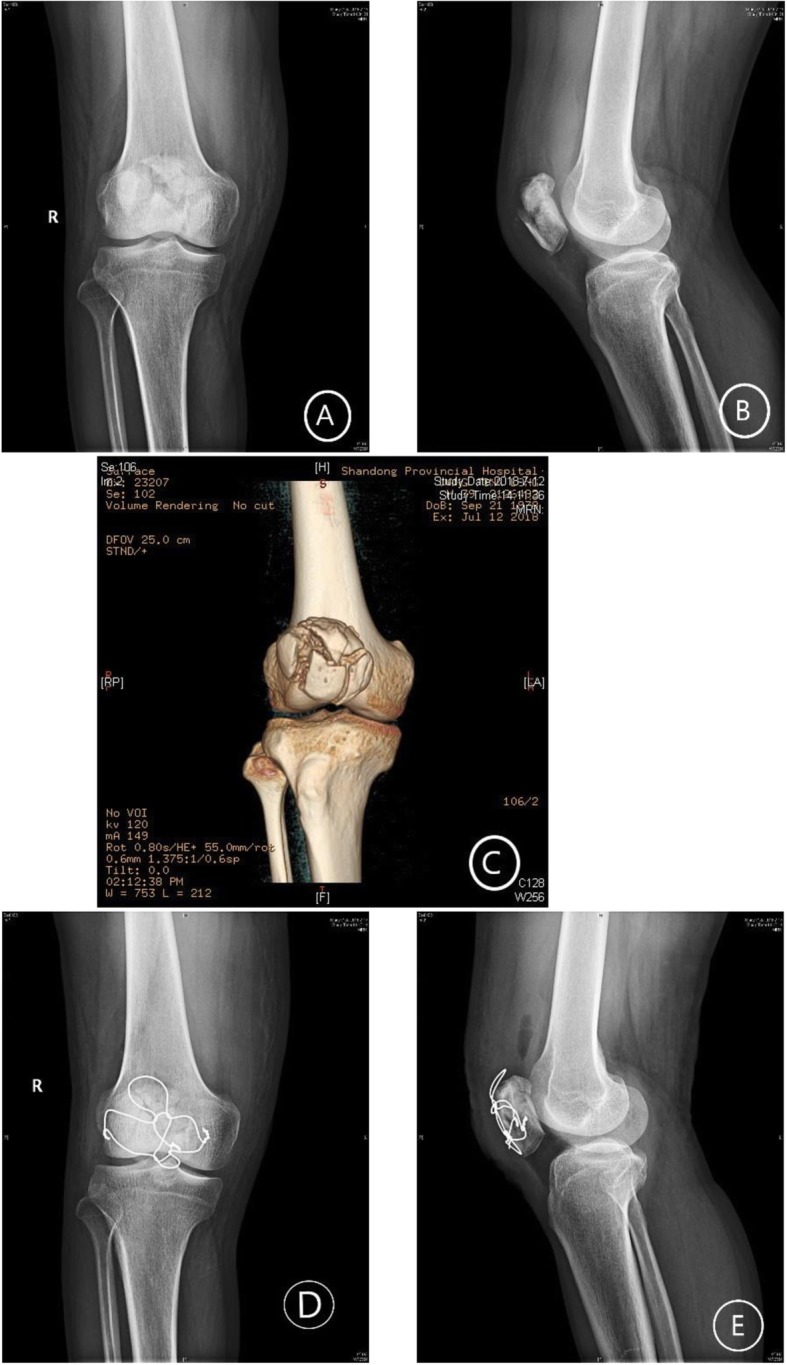
A 38-year-old male with severely comminuted patellar fracture. **a**, **b** X-ray before surgery. **c** CT before surgery. **d**, **e** Post-fixation with modified cerclage wiring

Under general anaesthesia or spinal anaesthesia, surgery was performed, and the supine position with the injury knee extended. The tourniquet was used. An anterior median incision of the knee joint was made. After incising the superficial fascia, the extensor apparatus was exposed and checked if destroyed. Then, the patella was completely exposed, and the fractured patella was presented. A haematon might be present and was derided. The fracture was reduced with reduction forceps and temporary fixation with towel clamp and Kirschner wires (K-wires) under direct vision. In some comminuted patellar fractures, more K-wires were required to penetrate the fracture line. The number of K-wires used in each patient depended on the type at the extent of the fracture. The articular surface was checked by an image intensifier. When the articular surface was smooth, the first stainless steel wire was sutured around half of the patella intermittently. During the suturing process, a number of steel wires were reserved. The second steel wire was sutured around the other half of the patella intermittently, and a number of steel wires were reserved. The third steel wire was used to penetrate the reserved steel wire in front of the patella. The third steel wire was tightened to the reserved steel wire segment, and the locking was fastened with uniform force. Then, the first and second steel wires on both sides of the patella were tightened by two surgeons simultaneously to an appropriate degree without overtightening, and the locking was fastened with uniform force (Figs. 1, 2 and 3). The image intensifier was used to check the articular surface again. The stability of fixation is assessed by the bending knee joint, and knee flexion is 90° to confirm that there is no separation between fracture fragments. The distal tip of each wire was trimmed and cut to avoid irritation of the soft tissue. Finally, the incision was flushed, the anterior patellar ligament was sutured and the incision was closed.

All patients were operated smoothly without blood transfusion and the operation time was 55–80 min (66.4 ± 7.1 min). 
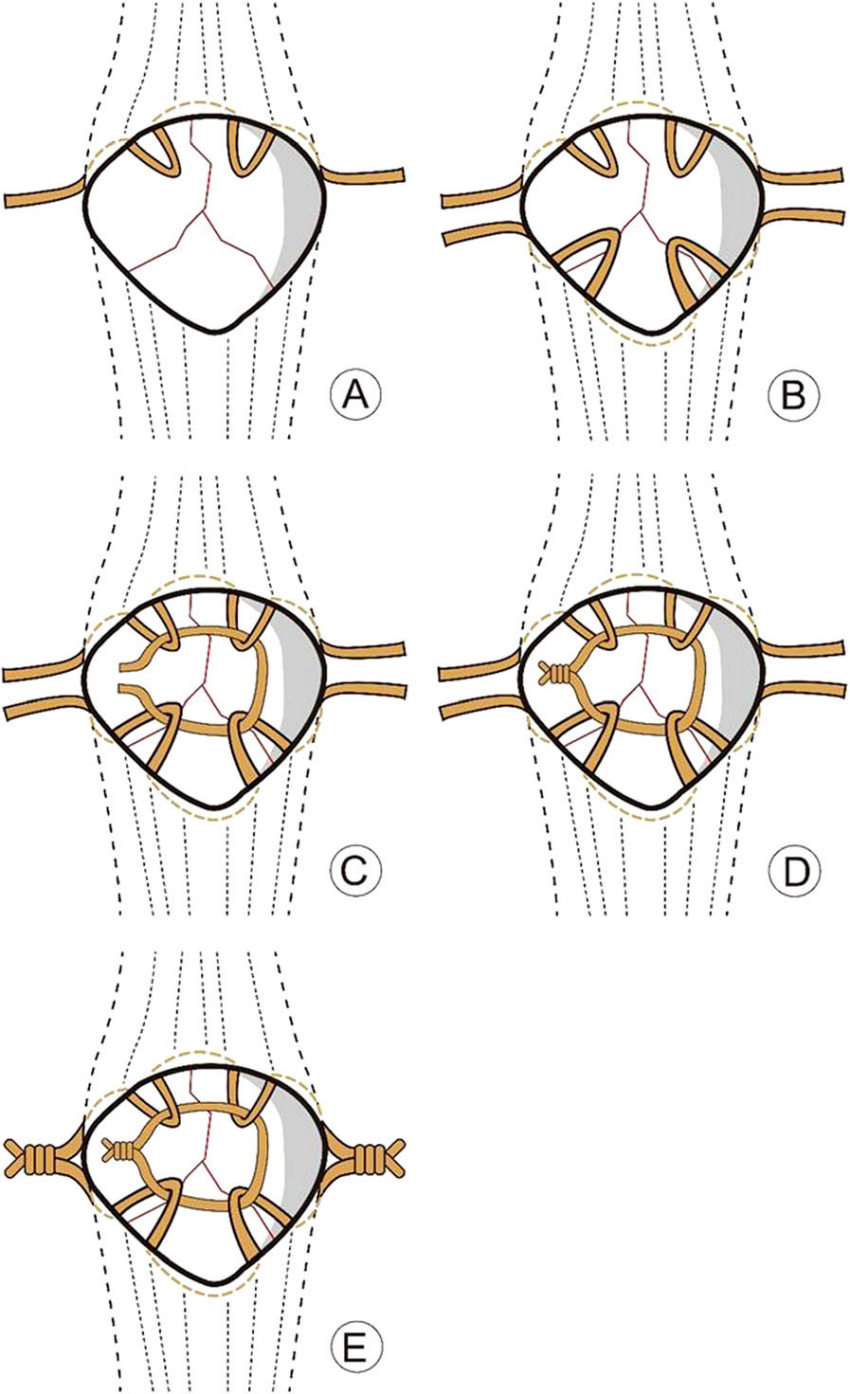


Illustrations of patellar fixation techniques using modified cerclage wiring fixation. (A) When the articular surface of the patella was smooth, the first stainless steel wire was sutured around half of the patella intermittently. During the suturing process, a number of steel wires were reserved. (B) The second steel wire was sutured around the other half of the patella intermittently, and a number of steel wires were reserved. (C) The third steel wire was used to penetrate the reserved steel wire in front of the patella. (D) The third steel wire was tightened to the reserved steel wire segment, and the locking was fastened with uniform force. (E) The first and second steel wires on both sides of the patella were tightened by two surgeons simultaneously to an appropriate degree without overtightening, and the locking was fastened with uniform force.

### Postoperative management

There is no need to fix the knee joint after the operation. Functional exercises were started with CPM on the first day after operation under the guidance of rehabilitation specialists. Patients were allowed to use crutches for partial weight-bearing and then complete weight-bearing at 4 weeks postoperatively. Posterior patellar images were obtained 2 days, 1 month, 2.5 months, 3 months, 6 months and 12 months after the operation. Criteria for fracture healing are as follows: no local pain or tenderness, good walking without help and trabecular bone growing along the fracture line with imaging evidence. During follow-up, the clinical grading scales of Böstman were used to evaluate the results after the operation from eight aspects: range of movement, pain, work, atrophy, assistance in working, effusion, giving way and stair-climbing. Among these scales, 28–30 points were excellent, 20–27 points were good and less than 20 points were poor [11].

## Results

Thirty-eight patients were followed up for 6–36 months (16.1 ± 5.8 months). The time from operation to fracture healing was 2.5–3.5 months (2.92 ± 0.25 months). There were no complications, such as loosening of internal fixation, bone re-displacement, nonunion, infection, breakage of internal fixation and traumatic osteoarthritis.

Using the clinical grading scales of Böstman, the average score of the final follow-up was 28.7 (range 20–30). The average score of 32 (84.2%) excellent patients was 29.5 ± 0.7 (range 28–30) and that of six (15.8%) good patients was 24.5 ± 2.2 (range 20–27). The range of knee flexion activity of patients was 130° (range 110–140), and the prognosis was satisfactory.

## Discussion

Surgical treatment of comminuted patellar fracture involving the articular surface is often complicated and difficult, mainly due to weak patellar bone and more small fragments. There are many problems in the treatment of circumferential cerclage wire fixation: due to the lack of fixed reliability, there are some problems in rehabilitation exercise, such as loosening and breaking of steel wire leading to the re-displacement of bone, failure of internal fixation and additional injury of the articular surface [2]. Cerclage wiring fixation is used as an adjunct with other fixation measures [16, 18]. However, Matsuo et al. [20] fixed five cases of patellar fracture with soft tissue around the bone at the same time. One case had distal nonunion but did not affect knee extension function. It is possible that in the treatment of comminuted fracture, the technique of encircling the soft tissue around the bone was superior to the traditional tension band technique. Tension band fixation using AO principles is the current standard of care and the most widely accepted method of fixation for displaced simple transverse fractures without significant comminution [21]. This technique alone is difficult to treat a comminuted patellar fracture. For complex patellar fracture patterns, tension band fixation with supplementation of interfragmentary screws or cerclage wires can achieve satisfactory results. Hambright et al. [22] described an enhancement to the traditional tension band construct that used additional wires and multiple tension bands to gather and fix comminuted fracture patterns, of which the clinical outcomes of 27 patients were satisfactory. Additional wires and multiple tension bands may severely damage the soft tissue around the fracture, and some complications, including wire migration, broken K-wires, pain from stainless steel wire loops and prominent hardware, may occur. We adopted a modified cerclage wiring fixation to treat a patellar comminuted fracture. We also call this technique “wire mesh”. The surrounding steel wire is sutured intermittently, and the surrounding ligament is repositioned indirectly. The tension around the patella is transformed into the pressure between the fragments of compression fracture. The third steel wire in front of the patella can eliminate the tension caused by knee flexion, provide a strong and stable fixation effect and enable the patients to perform early knee joint functional exercises. Avoiding joint stiffness may be an option for the treatment of comminuted patellar fractures. In our cohort study, 84.2% (32/38) of the patients with excellent results and 15.8% (6/38) of the patients with good results achieved satisfactory results, which were also applicable to the elderly with osteoporosis. In our study, the patients were given functional exercises early after the operation, and CPM-assisted functional exercises began on the first day after the operation, which may be one of the important reasons for the satisfactory results after the operation.

Lue et al. [17] used non-absorbable suture cerclage combined with nickel-titanium patellar concentrator to treat comminuted fracture patterns; 75.8% of the patients have excellent results and 24.2% have good results. These authors proposed that for comminuted patellar fractures, laterally displaced fragments cannot be stably fixed by the nickel-titanium patellar concentrator due to traction of the patellofemoral constructs during knee flexion. Wurm et al. [9] treated a patella fracture with an angular stable patella plate and have an overall complication rate of 6%. None of the patients reported any deficits in extension capabilities. The flexion was on average 127°. Matejcić et al. [23] evaluated a basket plate in the treatment of comminuted fractures of the distal pole of the patella and showed good results. We propose a new strategy of modified cerclage wiring fixation. Only three steel wires are used to fix the patella, which is easy to operate, low cost and less destruction to the blood supply in the fracture. It can provide stable fixation. Our study suggests that compared to other techniques, this new technique achieved similar clinical outcomes and had a low incidence of complications related to internal fixation.

The patella consists of a large number of cancellous bones, and the fracture heals quickly. The healing time of patellar comminuted fracture can be predicted if it can be fixed stably. In this study, the time from operation to fracture healing was 2.92 ± 0.25 months, which was similar to the average healing result of 2.81 months of comminuted patellar fracture reported by other scholars [17]. The patient factors directly affect the prognosis of patellar fracture after the operation. The risk of nonunion and infection in patients with a history of cerebrovascular accident increases by more than six times, and in diabetic patients, the incidence of secondary surgery increases by eight times [24]. Our study suggests that patients with a history of cerebrovascular accident or diabetes mellitus should carefully be considered.

In the course of clinical treatment, the authors consider that in the process of continuous suture of a steel wire, the flexibility of steel wire decreases and fatigue increases, which leads to a decrease in the toughness of the steel wire [25]. Therefore, we used several steel wires (mainly three) to suture and ring, which could not only reduce the change of the toughness of the steel wire itself but also be easy to operate and could resist the creep of the steel wire under certain tension. There was no loosening or breaking of steel wire. Of course, there are also shortcomings: requiring both sides to tighten the lock. Wu et al. [26] applied tension band wiring and had a measured knee joint flexion angle of 138.9° (110–140) after surgery. Chang et al. [27] applied tension band wiring through cannulated screws and had a knee joint flexion angle of 123° (100–140). Our patients, treated with modified cerclage wiring, had a knee joint flexion angle of 130° (110–140), suggesting that a similar range of motion to those reported was achieved with this new technique.

A recent study suggests that titanium cable cerclage should be used to treat displaced comminuted patellar fractures [8, 28]. In this technique, fracture fragments were fixed with K-wires, and titanium cable cerclage combined with K-wire fixation was used to maintain the reduction of bone and had good clinical results. Huang et al. [8] reported that only one patient suffered from titanium cable loosening 4 weeks after the operation. Additionally, the main defect of this technique was noted: if there were no K-wire fixation, the displacement of the titanium cable would easily occur. K-wire is also indispensable in the treatment of a patellar comminuted fracture. The role of K-wire changes complex comminuted fractures into simple fractures. Because of the poor stability of free fragments, especially comminuted fracture of the articular cartilage surface, the fragments are easy to displace and hard to achieve immediate and reliable fixation through simple towel clamp reduction. The aggregation and retraction force of the wire ring can often lead to fracture. End slip leads to failure of internal fixation, displacement of fracture fragments and the risk of loss of patellofemoral joint flatness. For some comminuted bones, we adopt modified cerclage wiring combined with K-wire fixation. When reducing fracture, the standard is an articular surface anatomical reduction. After reduction, K-wire is used to fix it first, and then steel wire is used to fix it, which makes the fixation more reliable. The number of K-wire depends on the degree of fragmentation.

In the treatment of comminuted patellar fracture with modified cerclage wiring, the author suggests that attention should also be paid to the following: (1) Choice of surgical incision: longitudinal incision or transverse incision? We chose a transverse incision because of convenient wire needle insertion and locking wire buckle. (2) Tighten the steel wire evenly to avoid overtightening or loosening. During the suturing process, we paid attention to avoid aggravating the displacement of the fracture block and preventing the wire around the patella from penetrating the block. We used the wire to go around the crushed pieces. (3) C-arm X-ray machine fluoroscopy should be performed many times during the operation to ensure satisfactory reduction. (4) During the operation, we should pay attention to protecting and repairing the patellar surface ligaments to avoid the formation of free bone fragments, use soft tissue to indirectly reposition the crushed bone fragments and avoid further peeling of the crushed bone fragments; at the same time, it can better protect the patellar periosteum and promote fracture healing. (4) To avoid the irritation of the tail end of the steel wire to the surrounding soft tissue, the tail of the steel wire should be trimmed and smoothed after locking and buried in the lateral soft tissue of the patella to avoid the discomfort caused by the irritation of the soft tissue.

The limitations of our study are the small sample size, the lack of a control group and the results may be biassed. Additional prospective and biomechanics studies should be conducted to confirm these outcomes in the future.

## Conclusions

Modified cerclage wiring is a new effective method for the treatment of a comminuted patellar fracture. This technique can provide strong and stable fixation, enable patients to perform an early functional exercise and has a good clinical effect.

## Abbreviation

K-wire: Kirschner wire
